# Supplementary material for: Glucose/Graphene-Based Aerogels for Gas Adsorption and Electric Double Layer Capacitors
Source: Polymers (Basel). 2018 Dec 28;11(1):40. doi: 10.3390/polym11010040 (PMC6401828; doi:10.3390/polym11010040)
Supplement: Supplementary file 1 [file polymers-11-00040-s001.pdf]

*Polymers* **2019**, *11*, 40; doi:10.3390/polym11010040

# Supporting information

# Glucose/Graphene-Based Aerogels for Gas Adsorption and Electric Double Layer Capacitors

**Kang-Kai Liu [1,†], Biao Jin [1,†], Long-Yue Meng [2,*]**

1 Department of Chemistry, Yanbian University, Park Road 977, Yanji 133002, China; lkk0391@163.com (K.K.L.); jinbiao@ybu.edu.cn (B.J.)
2 Department of Polymer Materials and Engineering, Department of Chemistry, MOE Key Laboratory of Natural Resources of the Changbai Mountain and Functional Molecules, Yanbian University, Park Road 977, Yanji 133002, China
† These authors contributed equally to this work.
* Correspondence: lymeng@ybu.edu.cn (L.Y.M.)

## Supplementary Figures

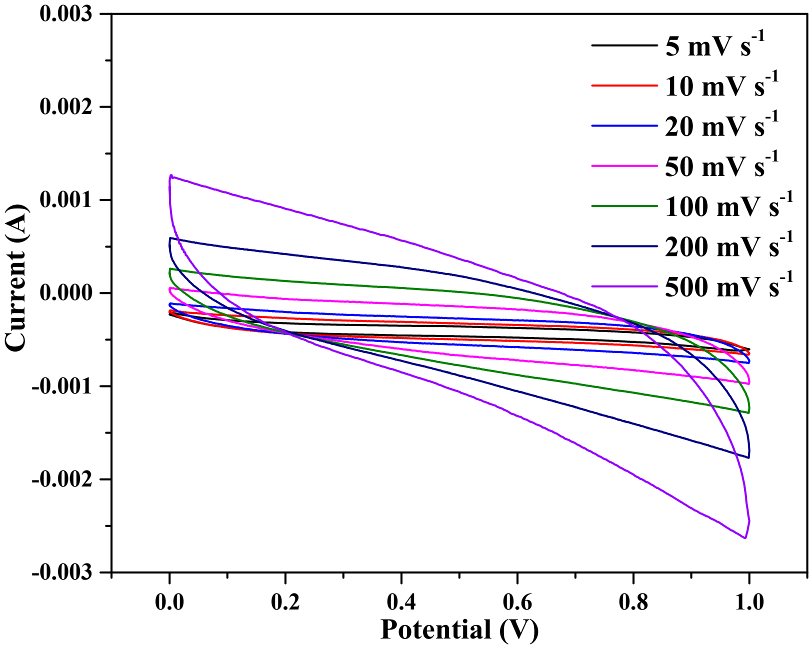


**Figure S1.** CV curves of HA-2 (4 mg $mL^{-1}$ glucose & 800 °C $CO_2$ activation) at different scan rates.

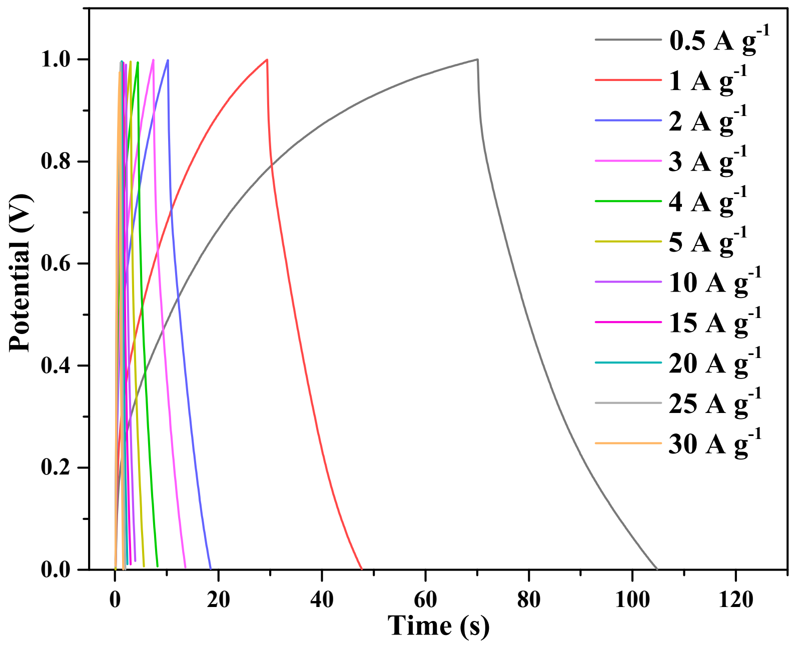


**Figure S2.** GCD curves of HA-2 (4 mg mL$^{-1}$ glucose & 800 °C $CO_2$ activation) at current densities.

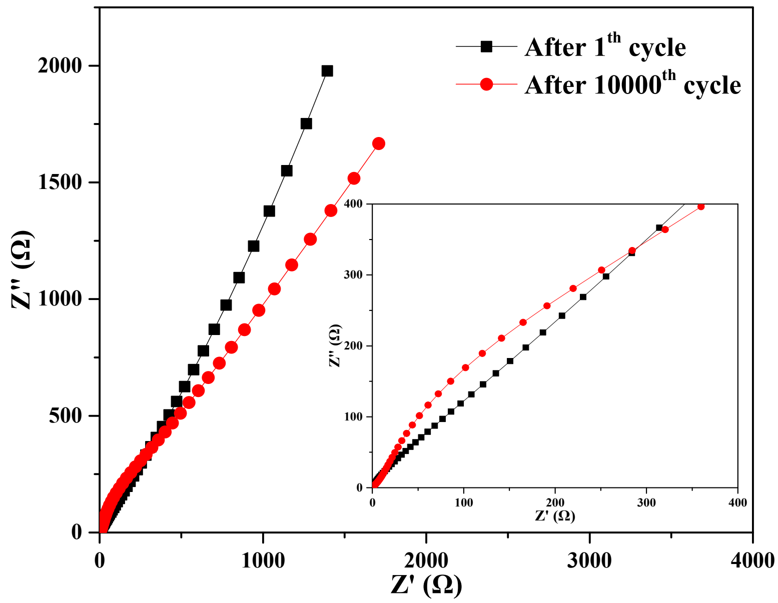


**Figure S3.** EIS curves of HA-2 (4 mg mL$^{-1}$ glucose & 800 °C $CO_2$ activation) after 1$^{th}$ cycle and 10 000$^{th}$ cycle.

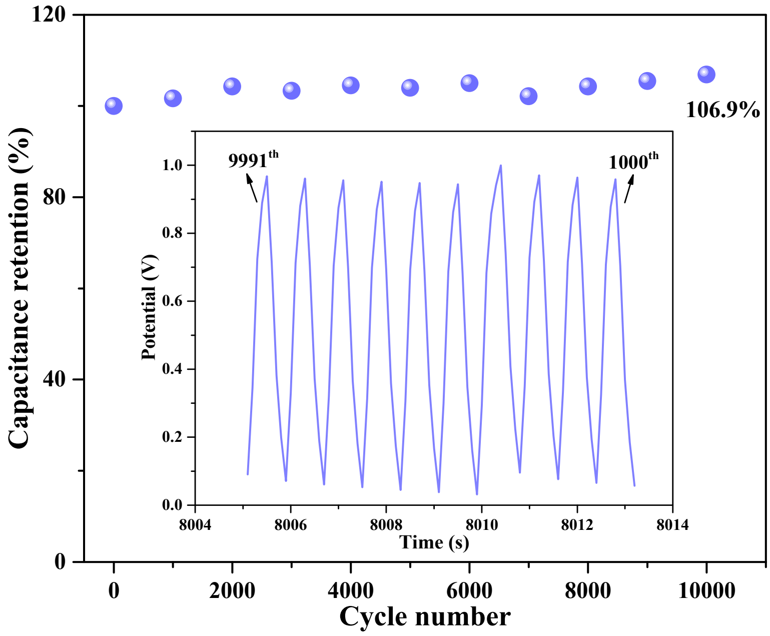


**Figure S4.** Cyclic stability of HA-2 (4 mg $mL^{-1}$ glucose & 800 °C $CO_2$ activation).

## Supplementary Discussion

To ensure the EDLCs device performances of the G/GAs based electrode, a symmetrical electrochemical capacitor device based on two G/GAs based electrode, as positive and negative electrodes, was measured in 6 M KOH. The CV curves of the device (Figure S1) keep more standard rectangular shapes than the single electrode, which exhibits the good stability of the charge transportation process. The triangles from GCD curves (Figure S2) indicate that the G/GAs based electrode exists the certain internal resistance, which results in the no linear dependence on the applied potential during the charging process, and the specific capacitance of 135.2 F $g^{-1}$ was obtained at 1 A $g^{-1}$. The Nyquist plots of HA-2 (Figure S3) presented no semicircle in the

high frequency region, but after 10 000 cycles, the line is closer to 45° than before, which indicates the mixed control between charge transfer and diffusion processes. Furthermore, according to the specific capacitance retention after 10 000 cycles at 10 A $g^{-1}$ in Figure S4, the wettability of G/GAs based electrode has increased in the charge-discharge process.

## Supplementary Reference

1. Hongshuai, G.; Bing, D.; Jie, W.; Yadi, Z.; Xiaodong, H.; Langyuan, W.; Yufeng, A.; Hui, D.; Xiaogang, Z. Template-induced self-activation route for nitrogen-doped hierarchically porous carbon spheres for electric double layer capacitors. *Carbon* **2018**, *136*, 204–210.
2. Tolendra, K.; Tran, D.T.; Soram, B.S.; Nam, H.K.; Joong, H.L. Hierarchical material of carbon nanotubes grown on carbon nanofibers for high performance electrochemical capacitor. *Chem. Eng. J.* **2018**, *345*, 39–47.
